# Toward Sustainable Xanthan Gum Production: Waste-Derived Substrates, Fermentation Optimization, and Eco-Friendly Extraction Approaches

**DOI:** 10.3390/foods15061100

**Published:** 2026-03-20

**Authors:** Peer Mohamed Abdul, Setyo Budi Kurniawan, Rosiah Rohani, Nor Sakinah Mohd Said, Rozieffa Roslan, Muhammad Fauzul Imron

**Affiliations:** 1 Department of Chemical and Process Engineering, Faculty of Engineering and Built Environment, Universiti Kebangsaan Malaysia, Bangi 43600, Selangor, Malaysia; peer@ukm.edu.my (P.M.A.); rosiah@ukm.edu.my (R.R.); norsakinahsaid95@gmail.com (N.S.M.S.); rozieffa.roslan@gmail.com (R.R.); 2 Research Centre for Sustainable Process Technology (CESPRO), Faculty of Engineering and Built Environment, Universiti Kebangsaan Malaysia, Bangi 43600, Selangor, Malaysia; 3 Centre of Excellence for Biomass Utilization, Universiti Malaysia Perlis (UniMAP), Arau 02600, Perlis, Malaysia; 4 Research Center for Environment and Clean Technologies, National Research and Innovation Agency (BRIN), Jakarta Pusat 10340, Indonesia; setyo.budi.kurniawan@brin.go.id; 5 Study Program of Environmental Engineering, Department of Biology, Faculty of Science and Technology, Universitas Airlangga, Kampus C UNAIR, Jalan Mulyorejo, Surabaya 60115, Indonesia; 6 Research Group of Sustainable Environmental Systems and Infrastructure (SUSTAIN), Faculty of Science and Technology, Universitas Airlangga, Kampus C UNAIR, Jalan Mulyorejo, Surabaya 60115, Indonesia; 7 Department of Water Management, Faculty of Civil Engineering and Geosciences, Delft University of Technology, Stevinweg 1, 2628 Delft, The Netherlands

**Keywords:** extraction, fermentation, sustainability, waste valorization, xanthan gum

## Abstract

Sustainable xanthan gum (XG) production is increasingly prioritized as global demand rises, and conventional processes face economic and environmental constraints. Traditional manufacturing depends heavily on refined sugars, intensive fermentation control, and solvent-based purification, which elevate production costs and ecological impact. This review highlights recent advancements designed to improve sustainability across the XG value chain, focusing on alternative substrates, optimized fermentation, and greener extraction methods. Agricultural residues, food-processing waste, lignocellulosic biomass, and industrial effluents have emerged as promising low-cost substrates that reduce reliance on refined sugar sources while supporting waste valorization. Pretreatment strategies, such as acid hydrolysis, enzymatic processing, and integrated biological–chemical methods, significantly enhance the accessibility of complex biomass for microbial fermentation. Concurrently, improvements in strain selection, metabolic engineering, and process control have increased XG yield, molecular weight, and rheological performance. Environmentally friendly extraction technologies, including ultrasound-assisted extraction, pulsed electric fields, membrane filtration, and electro-dewatering, further reduce solvent consumption and energy demand in downstream processing. However, challenges persist, including substrate variability, formation of inhibitory compounds, strain instability, and regulatory considerations for waste-derived substrates or genetically modified strains. Future progress will rely on integrating bioprocess intensification, genetic engineering, and techno-economic assessment to build scalable, low-impact, and circular XG production systems.

## 1. Introduction

Xanthan gum (XG) is an extracellular polymeric substance (EPS) mostly produced by the bacteria species of *Xanthomonas* sp. from the fermentation of carbohydrate-containing substrates [1]. The United States Department of Agriculture’s Northern Regional Research Center made the discovery of XG in 1963. Structurally, it is composed of a β-D-glucose backbone and trisaccharide side chains that contain mannose and glucuronic acid [2]. These side chains are responsible for the high molecular weight and distinctive rheological properties of the mixture. As a result of its extraordinary stability, pseudoplasticity, and viscosity across a broad spectrum of pH and temperature conditions, XG has emerged as one of the most adaptable microbial polysaccharides in commercial applications [3]. Early 1964 marked the commencement of substantial commercial production.

XG’s multifunctional properties have made it a popular choice in a variety of industries. In food systems, XG functions as a versatile hydrocolloid that provides viscosity, suspension stability, and emulsion stabilization at low concentrations [4]. Its strong shear-thinning behavior is particularly advantageous in salad dressings, sauces, and beverages, where it enables easy pouring while maintaining stability during storage [5]. In gluten-free formulations, XG mimics the viscoelastic properties of gluten by forming a hydrated network that improves dough handling, gas retention, and final product texture [4]. It is also widely used in dairy alternatives and reduced-fat foods to enhance mouthfeel and prevent phase separation without increasing caloric content [6]. Furthermore, XG contributes to shelf-life extension by inhibiting syneresis and stabilizing dispersed phases [7]. XG is also employed in the pharmaceutical and cosmetic industries for drug delivery systems, topical formulations, and personal care products as a result of its gel-forming capabilities and biocompatibility [8]. It is also employed as a viscosity modifier in drilling fluids and enhanced oil recovery procedures by the petroleum and oilfield sector [9]. Furthermore, its industrial relevance is further underscored by its implementations in textiles, agriculture, and biodegradable films [10].

The growing global demand for XG, which is driven by its expanding applications and the growing preference for biobased materials, requires a transition to more sustainable production models. The competition between industrial and food uses is frequently raised by the use of high-purity substrates, such as glucose and sucrose, that are derived from food-grade commodities [11]. From an economic perspective, downstream processing is one of the most cost-intensive stages of XG production due to the high solvent demand for precipitation, subsequent solvent recovery, and energy-intensive drying. The reliance on refined sugars as carbon sources further increases raw material costs [12]. Environmentally, these steps contribute to high water use, volatile organic solvent emissions, and elevated energy consumption, resulting in a larger carbon footprint [13]. Developing low-solvent, low-energy recovery methods and utilizing waste-derived substrates are critical for reducing both production costs and environmental impact [14,15,16].

Research has increasingly concentrated on the identification of alternative, low-cost, and renewable substrates for fermentation, including agricultural residues, industrial by-products, and food processing refuse, in response to these concerns [11]. Concurrently, novel extraction and purification techniques are being investigated in order to mitigate environmental impact, enhance yield, and reduce energy consumption [17]. These innovations are consistent with global initiatives to promote circular economy principles and minimize the ecological impact of biopolymer production.

Despite the extensive body of literature on XG production, most previous reviews have focused primarily on either its physicochemical properties and applications or on conventional fermentation using refined substrates and solvent-intensive downstream processing. Although several studies have reported the use of alternative carbon sources [18,19], these discussions are often fragmented and lack an integrated assessment of their implications for process sustainability, scalability, and environmental performance. In addition, recent advances in green extraction technologies, bioprocess intensification, and waste-derived feedstocks have not yet been systematically synthesized within a unified sustainability framework. Consequently, a comprehensive evaluation that simultaneously links substrate valorization, fermentation optimization, and eco-friendly recovery strategies is still lacking.

This review addresses this gap by providing an updated and holistic analysis of sustainable XG production across the entire value chain. Specifically, it (i) compares conventional and waste-derived substrates from a techno-environmental perspective, (ii) critically evaluates emerging low-solvent and low-energy extraction approaches, and (iii) highlights the integration of process optimization, genetic engineering, and circular economy principles for scalable implementation. By consolidating these dimensions, this review offers a distinct contribution beyond prior XG reviews and identifies key research directions toward industrially viable and environmentally responsible production systems.

## 2. Overview of XG Biosynthesis

### 2.1. Structure of XG

**Figure 1:**
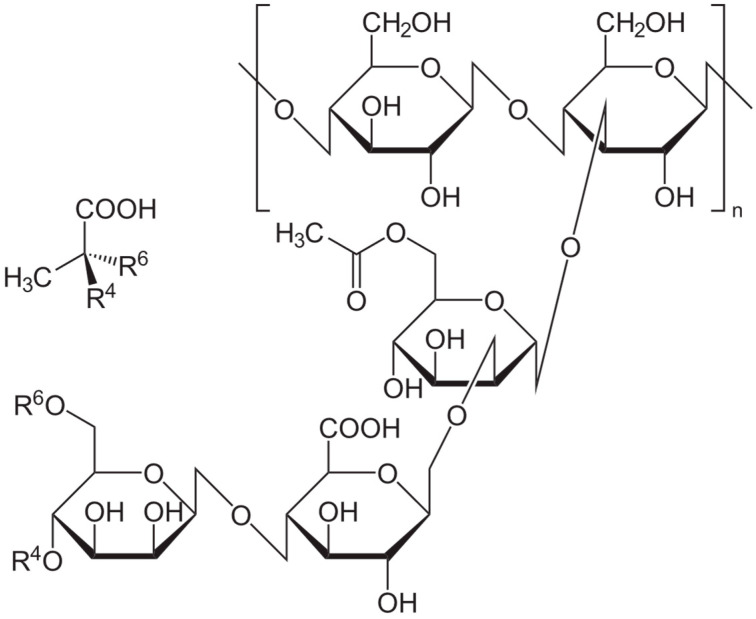
Chemical structure of xanthan gum.

XG is a heteropolysaccharide that is composed of a long chain of repeated pentasaccharide units of glucose, mannose, and glucuronic acid in the molar ratio of 2:2:1 [7]. The main chain is composed of β-D-glucose units that are linked at the 1 and 4 positions, resembling the cellulose structure [20] (Figure 1). The trisaccharide side chain is composed of a D-glucuronic acid unit between two D-mannose units that are linked to the alternate glucose unit in the main chain [7]. The D-mannose unit that is affixed to the main chain is linked to an acetyl group, while the terminal D-mannose unit is linked to a pyruvic acid residue [21]. The anionic nature of XG is attributed to the presence of acetic acid and pyruvic acid moieties [20]. The stability of XG is influenced by the primary structure of the XG chains and the type of functional group at the outer mannose unit (acetyl or pyruvyl) [22]. A higher degree of acetylation results in greater stability [23]. The concentration of pyruvic acid and acetyl is also contingent upon the microbial species employed and other fermentation conditions [24]; for example, industrial strains *X. campestris* NRRL B-1459 and ATCC 13951 [25,26] are known to produce xanthan with relatively high acetyl and pyruvyl content, which contributes to enhanced thermal stability and viscosity. The production of pyruvic acid moieties will be increased in the media that has been supplemented with citric acid [2]. Thus, the viscosity of solutions is significantly influenced by the pyruvyl concentration, which is a critical factor in a variety of food applications [27]. Gel formation necessitates a higher concentration of pyruvate, whereas solutions with a low concentration are less viscous.

The microbial strain, media components, operation conditions, and conformational changes all influence the content of the functional groups in the XG molecule [28]. The degree of substitution for pyruvate is typically 30–40%, while that for acetyl groups is 60–70% [29]. The native structure of XG undergoes a transformation to a disordered conformation as a result of thermal treatment of the solutions [30]. XG generates solutions that exhibit pseudoplastic or shear-thinning characteristics and are non-Newtonian [31]. Consequently, the solution becomes less viscous as the shear rate increases [32]. The shear-thinning behavior of xanthan gum solutions is governed by the transition between an ordered, rigid double-helical conformation and a disordered, flexible coil structure. At low shear rates, XG molecules adopt an ordered helical conformation that forms an entangled network, resulting in high apparent viscosity. Otherwise, these helices align in the direction of flow and partially unwind into more extended conformations [33], reducing intermolecular interactions and causing a marked decrease in viscosity. Higher molecular weight, greater pyruvylation, and increased chain rigidity enhance network formation and therefore intensify shear-thinning behavior, whereas lower molecular weight or reduced side-chain substitution leads to weaker intermolecular associations and a less pronounced pseudoplastic response [34]. This flow-induced structural alignment facilitates pumping and dispensing while maintaining high viscosity at rest, which is advantageous for flavor retention and mouthfeel in food systems [9].

### 2.2. Production of XG

Fermentation, biomass removal, product purification, and isopropanol recovery are the four primary stages of commercial XG production [4]. *Xanthomonas campestris* is initially cultivated in a fermenter that is well-agitated and aerated for the production process. A carbohydrate source, such as glucose or sucrose, a suitable nitrogen source, and nutrient ions are all included in the medium. The XG yield and structure are contingent upon the operational and environmental conditions [18]. Several factors, such as the type of fermenter used [35], operation mode (batch or continuous) [36], and dissolved oxygen concentration [37], influence the proliferation of bacterial cells and XG production. The production is also influenced by the concentration of medium components, particularly carbon and nitrogen [38]. In addition to carbon and nitrogen sources, the fermentation medium contains several inorganic salts and trace elements that are essential for cell growth and XG biosynthesis. Commonly used components include phosphate salts (KH_2_PO_4_ and K_2_HPO_4_), which act as buffering agents to maintain pH stability [39], and magnesium sulfate (MgSO_4_·7H_2_O), which serves as a cofactor for enzymes involved in nucleotide sugar synthesis [40]. Calcium ions (Ca^2+^) are often added to improve cell membrane stability and influence broth rheology [41], while iron (Fe^2+^/Fe^3+^) functions in electron transport and metabolic enzyme activity [42]. Trace elements such as Zn^2+^, Mn^2+^, and Cu^2+^ are required in small amounts for enzymatic reactions and cellular redox balance [43].

**Figure 2:**
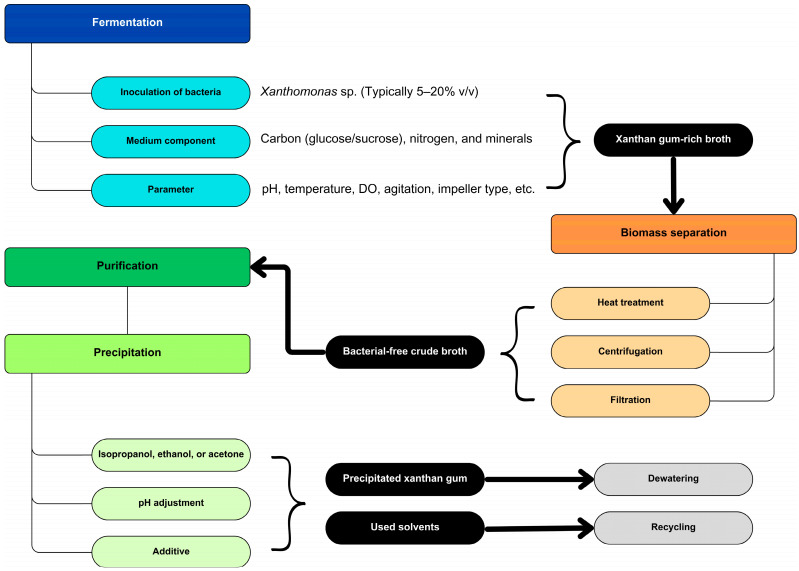
Schematic representation of the conventional xanthan gum production and recovery process consists of fermentation, biomass separation, and purification stages.

The processing parameters, such as inoculum size, temperature, pH, agitation, aeration rate, and the type of impeller used [44,45,46], are also important. Upon the completion of the fermentation process, the production medium contains the XG, microbial residues, and residual supplemented chemicals. The bacterial organisms are initially eliminated through heat treatment or alternative methods such as centrifugation or filtration [9]. In order to facilitate further purification, precipitation is typically conducted using organic solvents such as acetone, isopropanol, and ethanol, with the addition of specific compounds and pH adjustments [47]. The product is mechanically dewatered, dried, and packaged following precipitation [9]. Figure 2 illustrates a flowchart that delineates the procedures associated with the production of commercial XG.

### 2.3. Microbial Strains and Genetic Aspects

The production of XG is primarily associated with *X. campestris*, a Gram-negative bacterium whose genome encodes the complete set of genes required for the synthesis, polymerization, and export of this exopolysaccharide [48]. Central to this process is a well-defined gene cluster responsible for XG biosynthesis, which includes genes encoding *glycosyltransferases*, proteins for side chain modification, and enzymes involved in the terminal steps of polymer assembly and secretion [49,50,51]. At the genetic level, XG biosynthesis in *X. campestris* is primarily governed by the gum gene cluster (*gumB*–*gumM*), which encodes the enzymes and transport proteins responsible for the assembly [52], modification, polymerization, and export of the repeating pentasaccharide unit [53]. The early steps of repeat-unit assembly occur on a lipid carrier at the cytoplasmic membrane. *GumD* functions as the initiating *glycosyltransferase* that transfers glucose-1-phosphate to the lipid carrier, forming the first activated intermediate [54]. Subsequent sequential sugar additions are catalyzed by *GumM*, *GumH*, *GumK*, and *GumI*, which incorporate glucose, mannose, and glucuronic acid residues to complete the pentasaccharide repeat unit [55].

Side-chain modifications that determine the rheological properties of xanthan are mediated by *GumF*, *GumG*, and *GumL*, which catalyze acetylation and pyruvylation reactions on the terminal mannose residues [56]. These non-carbohydrate substitutions strongly influence molecular conformation, viscosity, and gel-forming behavior. The polymerization and translocation of the repeat units across the membrane are controlled by *GumE*, a Wzy-type *polymerase* responsible for chain elongation [57], and *GumB* and *GumC*, which form part of a membrane-associated export complex analogous to the Wza/Wzc-dependent polysaccharide secretion system [58].

In addition to the core gum operon, genes involved in nucleotide sugar precursor synthesis, such as those encoding UDP-glucose *pyrophosphorylase*, GDP-mannose *dehydrogenase*, and UDP-glucuronic acid biosynthetic enzymes, play a critical role in controlling flux toward xanthan production [59,60]. These genes are located outside the gum cluster and connect central carbon metabolism with exopolysaccharide biosynthesis. Regulation of the gum operon is influenced by global transcriptional regulators, carbon source availability, and dissolved oxygen levels [61], which together modulate precursor supply and polymer yield.

From a metabolic standpoint, *X. campestris* utilizes multiple pathways for glucose catabolism. The predominant route is the Entner–Doudoroff (ED) pathway [62], which operates alongside the tricarboxylic acid (TCA) cycle to generate energy and metabolic precursors. A smaller fraction of glucose is processed through the pentose phosphate pathway (PPP), which contributes to NADPH production and biosynthetic intermediate supply [63]. The presence of two distinct systems for glucose uptake underscores the bacterium’s metabolic flexibility and its adaptation for efficient polysaccharide production under varying environmental and nutritional conditions.

### 2.4. Fermentation Process Fundamentals

The fermentation process commences with the introduction of xanthan-producing bacteria, primarily *Xanthomonas* sp., and a nutrient-rich medium into the bioreactor [64]. As a carbon source for the microbes, this medium typically contains glucose, sucrose, or other fermentable sugars [65]. The majority of industries typically favor batch culture fermentation over continuous processes. The bioreactor’s abiotic environment is meticulously managed, with specific parameters such as temperature (28–30 °C), pH (7–8), oxygen levels (1 L/min), and agitation rate (200–300 rpm initially, with a subsequent increase to 400–600 rpm) being closely monitored [66]. This establishes an optimal environment for the bacteria to flourish. The bacteria undergo metabolic processes to multiply and produce XG as they consume the sugars. The bacteria excrete XG as an extracellular polysaccharide, which forms a viscous, gel-like substance [7]. Naturally, XG serves as the bacteria’s protective barrier, enabling them to endure in challenging environments [67]. The XG is extracted when the fermentation reaches its apex. The bacterial biomass is typically separated from the XG solution by centrifuging or filtering the mixture in the bioreactor [6], as depicted in Figure 2.

## 3. Factors Influencing XG Yield and Quality

### 3.1. Microorganism Species

The sugar sequence, linkages, and functional group incorporation of the polysaccharide are all influenced by the microorganism’s strain, which determines the XG production to a greater extent [7]. The selection of the microbial strain plays a critical role in determining the yield, viscosity, molecular structure, and overall quality of XG. Among various species within the *Xanthomonas* genus, *X. campestris* is the most widely used and industrially validated organism for commercial XG production [68]. However, several other strains, including *X. pelargonii* [69], *X. axonopodis* [70], and *X. phaseoli* [62], have also demonstrated potential in laboratory and pilot-scale studies, exhibiting differences in productivity, sugar utilization profiles, and XG properties.

Each strain possesses unique genetic and metabolic characteristics that influence XG biosynthesis. These differences can result in variations in monosaccharide composition and degree of pyruvylation and acetylation of the XG [28], molecular weight distribution [71], and the rheological behavior of the final product [6]. In addition, strain-dependent factors such as tolerance to substrate inhibitors, oxygen transfer efficiency, and fermentation kinetics directly impact fermentation performance and product consistency [18].

Recent advances in strain screening and genomic analysis have enabled researchers to identify novel *Xanthomonas* strains with enhanced XG production capabilities or modified product characteristics [72]. For example, mutant strains or genetically engineered variants of *X. campestris* have been developed to improve substrate utilization, reduce by-product formation, and tailor the functional properties of XG for specific industrial applications, such as producing white XG with reduced impurities and less solvent need during extraction, increasing yield as compared to wild-type, and producing less-viscous XG [73,74,75]. Production of XG under various conditions is tabulated in Table 1.

### 3.2. Carbon Source

Carbon source is one of the macronutrients that are necessary for the cell proliferation of microorganisms during the production of XG [18]. The primary commercial carbon sources for large-scale XG production are glucose and sucrose [82]. The production of XG is inhibited by a carbon source concentration that is either too low or too high [82]. The optimal carbon concentration for XG production is approximately 2–4%. The most common choice for the production of XG is sucrose. Other commercial substrates, including galactose, lactose, and xylose, may also be employed; however, the yield is low due to the bacteria’s incapacity to employ these substrates. In media that contain lactose as the carbon source, *X. campestris* produces a minimal level of XG due to the absence of the enzyme β-galactosidase, which ferments lactose. According to previous research, the production of XG is believed to be improved by a high carbon-to-nitrogen ratio (C/N) [83]. Sucrose yielded 13.234 g/L, while glucose yielded the maximum yield of 14.744 g/L of XG [84]. *Xanthomonas* sp. utilizes the Entner–Doudoroff pathway in conjunction with the tricarboxylic acid (TCA) cycle pathway to metabolize glucose [85].

### 3.3. Nitrogen Source

Organic and inorganic nitrogen sources are essential macronutrients for submerged fermentation of XG production [4]. In contrast to inorganic ones, organic ones are less costly. Inorganic sources may be derived from ammonium or nitrate salts, while organic sources may include peptone, yeast extract, maize steep liquor, and soybean meal. Peptone and yeast extract, which are common organic nitrogen sources, are the most suitable for the production of XG [4]. Ammonium compounds are regarded as a superior substrate for biomass accumulation among inorganic sources, while nitrate is the preferred option for achieving the highest XG yields [86]. As per the previous report, a steady carbon-to-nitrogen ratio must be maintained during the XG production and cell growth phases in order to achieve the highest XG production [87]. A high concentration of nitrogen sources is indicative of a high biomass yield. Nevertheless, numerous studies have demonstrated that the extremely high nitrogen source concentrations that are employed for enzyme production and cell proliferation are not suitable for the production of XG [87]. In the initial phases of the fermentation process, high nitrogen levels facilitated rapid cell growth, whereas later in the process, nitrogen levels are permitted to decrease [88].

### 3.4. pH

The pH has a significant impact on the production of XG, as it alters the charge density of the XG, which in turn alters the molecular association between XG molecules [89]. This results in a variation in the viscosity of the XG [90]. The optimal pH for *X. campestris* growth is between 6 and 7.5, while the optimal pH for XG production is between 7 and 8 [91]. The microorganism’s growth was observed to be facilitated by controlled pH, while XG production was not significantly affected [87]. Viscosity of XG solutions was observed to be unaffected within the pH range of 1–13. However, at a basic pH of 9 or higher, XG is observed to lose acetyl groups. Conversely, at a pH below 3, XG loses the pyruvic acid acetyl groups [87]. Alkali stress induces an increase in XG production as a protective mechanism in adverse conditions [90].

### 3.5. Temperature

The efficient production of XG is also significantly influenced by temperature. The production is primarily influenced by temperature fluctuations that occur during the proliferation of fermenting microorganisms. It was reported that the optimal temperature for the growth of microorganisms and the production of XG exhibits a minor variation, with *X. campestris* growing optimally at 25–27 °C and XG being maximally produced at 25–30 °C [92,93,94]. The structural composition is influenced by the temperature gradient during cultivation, resulting in a transition from an ordered to an unorganized state. Consequently, the viscosity of the material is also altered [95]. However, an additional increase in temperature may result in a reverse effect, which would reduce the production of XG and biomass [95]. The molecular conformation of XG is influenced by elevated temperatures to approximately 34 °C during the production of XG. This results in XG with a low acetate and pyruvate content and a low average molecular weight, which in turn results in low-viscosity aqueous solutions [28]. XG with a high acetate content and a high average molecular weight was synthesized at a low temperature of 25 °C, resulting in solutions with a high viscosity [28].

### 3.6. Agitation Speed and Aeration Rate

The agitation speed and aeration rate are of high importance in the production of XG, as *Xanthomonas campestris* is an aerobic microorganism. The oxygen mass transfer rate decreases as XG production increases as a result of the extracellular deposition of XG [28]. Additionally, the uniformity and nutrient distribution of culture media are disrupted as a result of an increase in viscosity, which in turn reduces the aeration rate [96]. The production of XG is proportional to the dissolved oxygen rate, as demonstrated by numerous investigations. *Xanthomonas*’ aerobic nature necessitates a high oxygen level in the media to facilitate the efficient activity of microorganisms and, as a result, increased XG production [97]. Moreover, the hydrodynamic stress may cause cell breakdown, resulting in a decrease in XG production [98,99]. The issue can be resolved by optimizing the mixing conditions [100,101]. Referring to Table 1, high productions of XG were achieved with an agitation rate ranging from 180 to 600 rpm, maintaining the oxygen transfer and avoiding shear stress. Jadhav et al. [102] have effectively implemented a novel fermentation technique in the laboratory that utilizes hydrogen peroxide as an oxygen source to surmount the resistance to gas–liquid mass transfer in the XG fermentation medium.

### 3.7. Fermentation Conditions Operation Mode

The time required for the fermentation process is directly proportional to the availability of substrate to be converted during the process and the dynamics of cell proliferation [103]. The average molecular weight of XG, as well as the concentration of pyruvyl and acetyl, tends to increase as the fermentation time increases [7]. Continuous fermentation is the preferred method to optimize production synthesis and fermentation time, as the process concludes upon the completion of substrate consumption and the onset of the cell decline phase [103]. A high XG yield can be obtained independent of fermentation time by optimizing other fermentation conditions, such as the bioreactor, strain, and substrate type used [104].

XG production can be achieved through either batch or continuous fermentation. The substrate to XG conversion efficiency is high (75–80%) in batch-scale fermentation; however, the process requires more than two days [4]. The increase in viscosity during this phase of the fermentation process may result in modifications to the accessibility of nutrients and the level of oxygen [105]. Continuous fermentation may serve as an alternative to the complications that may arise in a bulk process. A continuous flow of culture medium through the reactor is maintained in the subsequent process to ensure continuous nutrient availability and optimization. In continuous fermentation, the substrate to XG conversion rate is 60–70% [4]. Prasertsan et al. [106] conducted a study on the optimal parameters for exopolysaccharide production, and their findings indicated that batch cultivation yielded superior results in laboratory settings compared to continuous or fed-batch cultivation [95]. Continuous cultures are widely employed in industries due to their high substrate conversion rate and maximum cell production. However, they also have several drawbacks, such as the challenge of maintaining a constant setup for oxygen supply and the potential for contamination, which can result in a significant loss of product volume [95].

### 3.8. Stimulant

Citric acid has been discovered to increase the production of XG [90]. The productivity of XG is enhanced by the prevention of salt precipitation during heat sterilization through the use of citric acid as a chelating agent. In most studies, citric acid is added at concentrations ranging from 2.1 to 5.0 g/L, with optimal stimulation of XG yield commonly observed around 1–3 g/L [107,108,109]. The addition of salts to the production media prior to thermal treatment can optimize stability and mitigate any negative impact on XG conformation [95]. The degree of XG pyruvilation is increased by an increase in nitrogen concentration, particularly when an organic nitrogen source is employed [89]. The addition of corn steep fluid at a rate of 1 g per liter not only enhances the yield and viscosity of the XG produced but also reduces the cultivation time and encourages the utilization of more sugar [110]. XG is soluble in acetic acid, a mild carboxylic acid, and its solubility in the solution can be enhanced by its addition.

## 4. Conventional Extraction and Purification Strategies

Traditionally, the intricate process of extracting XG from *Xanthomonas campestris* follows a series of straightforward steps, involving: (i) medium preparation; (ii) fermentation; (iii) separation; (iv) purification. While considering the factors affecting the respective process, the forefront selection of fermentation medium directly affects the yield, quality, and production cost of these processes. To support microbial growth and XG fermentation, the presence of essential nutrients, particularly carbon and nitrogen sources, is vital to ensure efficient biomass development and optimal XG production [111]. Glucose and sucrose are the most widely used carbon sources in XG fermentation because they can be rapidly transported into cells and directly metabolized through the Entner–Doudoroff pathway and the tricarboxylic acid cycle [4], generating the nucleotide sugar precursors required for polysaccharide biosynthesis. In contrast, more complex carbohydrates must first undergo extracellular or enzymatic hydrolysis to release fermentable monosaccharides, which can limit substrate utilization and reduce productivity. The preference for mono- and disaccharides therefore reflects their higher metabolic accessibility, lower enzymatic demand, and more efficient conversion into xanthan repeat units [4]. This has been recognized as one of the major limitations of conventional methods, thereby prompting the exploration for cheaper alternatives to nutrient sources for the bacteria. In terms of nitrogen source, research indicates that both organic and inorganic sources may be effectively utilized in the fermentation process. Among conventional organic nitrogen sources are urea, tryptone, bacto-peptone, and yeast extract; while examples of inorganic sources are ammonium sulfate/phosphate and potassium nitrate [19]. In most cases, organic sources give better efficiency than the latter, depending on the strain of bacteria used. Those traditional sources are effective, but their cost might be prohibitive.

The extraction process largely depends on both thermodynamic and kinetic factors. However, thermodynamically favored techniques often result in low yields, making it necessary to focus more on kinetic considerations. Therefore, the introduction of non-exhaustive microextraction techniques emphasizes improving the kinetic aspect, such as by incorporating stirring, since the efficiency of extraction heavily relies on the diffusion rate of analytes within the sample [112].

Following the fermentation, the XG-rich broth then undergoes separation and purification steps to remove the cellular debris, residual nutrients, and other impurities. Conventional separation methods involve removing the biomass through centrifugation or filtration, after which the XG-rich supernatant is collected and further subjected to alcohol precipitation. Alcohol precipitation is a process to aggregate and precipitate out XG as a fibrous material by exploiting the polysaccharides’ limited solubility in certain solvents. The most commonly used solvents are ethanol and isopropanol, while acetone is employed less frequently because of its higher volatility and safety concerns [55]. Among these, isopropanol is often preferred at an industrial scale because it provides higher precipitation efficiency at lower solvent-to-broth ratios and promotes faster polymer aggregation, leading to improved solid–liquid separation. Ethanol, on the other hand, is favored in food-grade applications due to its lower toxicity and regulatory acceptance, although it typically requires higher volumes to achieve comparable recovery.

The last step, purification, is a step of washing the XG to remove residual impurities and solvents. The drying step is often carried out using hot air ovens or vacuum drying systems to reduce its moisture content. The resulting XG is milled into powder and stored under controlled conditions to maintain quality. Overall, conventional extraction methods have certain limitations, such as high alcohol consumption, energy demand for drying, and moderate product purity. These challenges have driven recent research into more sustainable and efficient extraction techniques, as detailed in the following section. Recent advancements in XG extraction techniques have focused on improving sustainability, reducing operational costs, and enhancing product quality through greener and more efficient strategies.

## 5. Alternative Substrates for XG Production

The global population’s urbanization and evolving behaviors have resulted in a substantial increase in refuse production from a variety of domestic, agricultural, and industrial sources. According to the Food and Agriculture Organization (FAO), more than one-third of the food that is produced annually is discarded. Furthermore, the quantity of waste continues to increase, particularly in developed countries, despite the existence of stringent legislation on food waste reduction as part of the Sustainable Development Goal (SDG) 12. These wastes mostly contain high-quality carbon sources, including glucose, fructose, maltose, lactose, and numerous others, for the biosynthesis of EPS. Recent studies have been introducing the utilization of food and agricultural waste as alternative nutrient sources for this process. It contains a large amount of nutrients, which on the one hand, can cause environmental pollution, and on the other hand, can be useful as a nutrient medium. A study by Santos et al. [113] reports on the production of XG from cassava peels and produced water as a nutrient source by bacteria *Xanthomonas axonopodis*, yielding 6.80 g/L with emulsification indices more than 50%, comparable to the commercial XG. Meanwhile, an example of food waste consumption for this respective process had been presented by Cancella et al. [68], utilizing milk permeate and cheese whey as substrates, where both produced XG with high thermal decomposition resistance. Wastewater may also be used as substrate, which also serves as a sustainable solution for waste degradation and was able to reduce the nutrient concentration during the XG biosynthesis [114]. Ozdal et al. [19] present the potential of chicken feather peptone as a nitrogen source along with molasses as a carbon source, with XG production as high as 20.5 g/L after 20 h cultivation at 30 °C, pH 7, and 200 rpm agitation. Furthermore, *X. campestris* is virulent as a phytopathogenic bacterium as a result of its capacity to secrete extracellular enzymes, including cellulase (endoglucanase), pectinase, protease, and amylase [115]. These enzymes, when secreted during the fermentation process, could assist in the hydrolysis of biopolymers (polysaccharides and proteins) that are present in food and agro-waste, resulting in the formation of simpler molecules (monomers) that can be converted into XG [116].

Advancements in the fermentation process included the alternative of pre-treatment of the lignocellulosic biomass to enhance the sugar release, leading to higher XG yields. Jazini et al. [117] present a research work on the effectiveness of phosphoric acid pretreatment in enhancing XG yields from raw Cedar and Elm woods. Respective works present yields of 9.9 g and 10.4 g XG per 100 g of raw Cedar and Elm wood, respectively, which are 4.95 and 4.33 times higher compared to much lower yields from untreated woods at 2.0 and 2.4 g. The pretreatment temperature optimization is also one of the keys in improving extraction, where a lower temperature with shorter duration not only improves yields but also reduces cost related to the extraction process [117]. But it also means an increment in pretreatment cost; thus, economic analysis needs to be assessed to study its feasibility for implementation. By amending the utilization of those resources for the medium preparation, it is not only able to minimize raw material cost up to 70% but also integrates a novel appeal in integrating waste valorization, waste management, and sustainable approaches in XG production. With that being mentioned, this also aligns with the Sustainable Development Goals, especially SDG 12 (Responsible Consumption and Production), SDG 9 (Industry, Innovation and Infrastructure), and also indirectly contributes to SDG 13 (Climate Action) by lowering greenhouse gas emissions and carbon footprint. As mentioned by Rončević et al. [118], the cost for those resources is the major contributor to the manufacturing cost for cultivation media preparation.

Some advancements in the extraction technologies have been studied, namely ultrasound-assisted extraction (UAE), pulse electric fields (PEFs), and High Voltage Electrical Discharge (HVED). UAE applied ultrasonic waves to enhance the extraction process, thus reducing the need for high temperatures and large volumes of solvent [119]. It utilizes cavitation phenomena to enhance mass transfer, resulting in higher yields and reduced extraction times while preserving the integrity of sensitive compounds to maintain the quality of produced XG [120]. While PEF and HVED apply electrical pulses to disrupt cell membranes, which directly facilitates the release of XG. Atmospheric and room temperature plasma (ARTP) technology applied by Gan et al. [74] produced an X-20 mutant that yielded up to 30% more than the starting strain, with improved viscosity and molecular weight. Engineered bacteria have also proven to have the ability to reduce up to 133% of ethanol required during downstream processing, with an improvement in the whiteness of the XG [4].

**Figure 3:**
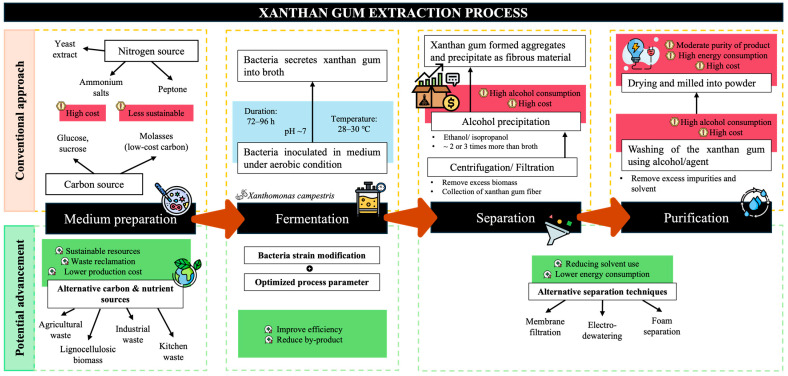
Conventional and potential advancement pathways for XG extraction and purification. The top part of the schematic (orange) represents the conventional downstream route. The bottom part (green) illustrates emerging and more sustainable alternatives and their benefits.

While alcohol precipitation remains the standard separation stage, recent studies have explored alternative separation techniques to minimize solvent use. These include membrane filtration, electro-dewatering, and foam separation, which aim to reduce both chemical input and energy consumption during the drying process. Utilizing membrane processes leads to higher purity levels in XG production due to its effectiveness in impurity removal, while also lowering the energy consumption as it operates at lower temperatures [121]. Electro-dewatering is a good alternative for moisture content reduction, thus helping in enhancing the drying process and overall yield [122]. Foam separation utilizes the adsorption of XG at the gas–liquid interface, allowing for the selective recovery of the XG through foam fractionation. This method is noted for its environmental friendliness and efficiency in separating surface-active agents from solutions [123]. A detailed comparison of advanced XG extraction options is tabulated in Table 2, while the overall representation of the advancement scenario is illustrated in Figure 3.

Besides, incorporating process optimization has also been reported to improve the efficiency of XG extraction. Real-time monitoring and control systems for parameters such as pH, dissolved oxygen, and nutrient concentration enhance fermentation consistency and allow fine-tuning of conditions to maximize XG yield. As mentioned by Zakeri et al. [125], factors that have a significant effect on XG yield are agitation rate, carbon source, and temperature, which may be optimized with the help of software such as Response Surface Methodology (RSM). Recent studies emphasize the integration of stirring in extraction processes to enhance yield by improving kinetic factors, which facilitates faster diffusion of analytes [111]. Techniques like RSM and artificial neural networks have been employed to optimize extraction parameters, leading to improved yields and efficiency [126]. Several alternative carbon and nutrient sources for XG production are tabulated in Table 3.

It is worth noting that the XG yield of 51.62 g/L obtained from jackfruit seed powder in Table 3 represents an outlier relative to most waste-based fermentations. This value was achieved under highly optimized medium conditions using RSM, with specific supplementation of peptone, phosphate salts, and citric acid, which functioned as nitrogen sources, buffering agents, and metabolic enhancers [90]. While the study confirmed the polymer identity using FTIR and XRD, the exceptionally high yield has not been widely replicated in subsequent independent studies, and most alternative substrates report substantially lower concentrations. XG production from food and agro-wastes generally obtains typical optimized yields around 30–40 g/L, even under controlled conditions [65]. This suggests that the jackfruit-based result may reflect a combination of aggressive medium optimization, strain-specific performance, and favorable nutrient supplementation rather than the intrinsic superiority of the substrate itself. Several alternative carbon sources for XG production are discussed below.

### 5.1. Agricultural Residues

Among the food and agro-industrial waste that have the potential and could be explored for XG production are fruit, seed, and peel extracts, molasses, lignocellulose, wastewater, and dairy wastes. In order to enhance their potential for XG production, each of these residues is subjected to a variety of valorization techniques. Furthermore, minerals and enhancers are incorporated into the purified waste to increase the yield of XG and the proliferation of cells [1]. These may consist of peptone and yeast extract to function as nitrogen sources, as well as mineral salts that could assist in pH maintenance, which are crucial mineral sources during the fermentation process [131]. Alternatively, the conversional method of utilizing alternative nitrogen sources could be implemented [19].

### 5.2. Industrial and Food Processing Waste

In the confectionery industry, in particular, a significant amount of effluent is produced, which is characterized by high levels of COD and BOD due to its abundance of readily biodegradable organic substances [132]. This makes it a prospective alternative for the preparation of media for bioprocessing. Using media containing confectionery wastewater, the highest yield of XG was reached at the end of fermentation at 10.03 g/L [133]. This resulted in a significant 66.85 % conversion of carbohydrates and 79.87 % conversion of sugar into the final product [133]. *X. campestris* ATCC was also employed to produce XG from wastewater from the wine industry. The resulting XG yields were 20.92 g/L and 30.64 g/L on media derived from white wine wastewater and rose wine wastewater, respectively [1]. Previous study also confirmed that XG production could be attained using a medium based on crude glycerol as the sole carbon source, with the use of various strains of *X. campestris*. Notably, the Xp 3–1 strain was employed to obtain the maximum concentration of XG (7.67 g/L) [65], while a mutant strain of *X. campestris* CCTCC M2015714 yielded 11.0 g/L [78].

### 5.3. Lignocellulosic Biomass and Pretreatment Needs

Lignocellulosic materials have recently garnered considerable attention as renewable and readily accessible resources for the production of a variety of bio-derived products, including XG. These lignocelluloses are derived from a variety of sources, including shells, hulls, and pulps. However, their resistant composition hinders microorganisms from directly utilizing them as a carbon source. Three primary stages are involved in the conversion of lignocellulosic materials into valuable products: pretreatment, hydrolysis, and fermentation [134,135]. During the initial phase, the complex structure of lignocellulosic materials is disassembled to improve the accessibility of cellulose for the hydrolysis process.

The use of *X. campestris* pv. *campestris* IBSBF 1866 and 1867 in the fermentation of cocoa husk as carbon sources (2 %, thermal treatment, 121 °C for 15 min), yielded 4.48 g/L and 3.89 g/L, respectively [136]. While the sago industry tapioca pulp (pre-treated with 0.5% sulphuric acid at varying concentrations (0.5 %, 2.5 %, and 5.0 %)) was used, the yield of XG by *X. campestris* NCIM 2954 was 7.1 g/L [137]. Fermentation of green coconut shell, passion fruit peel, corn straw, and corn cob (pretreated with sodium hypochlorite for 15 min and autoclaved at 121 °C for 15 min to undergo self-hydrolysis) by *Xanthomonas campestris* pv. *campestris* (1078) resulted in XG yield of 5.5 ± 3.8, 6.7 ± 1.9, 1.0 ± 0.04, and 2.7 ± 0.1 g/L, respectively [138]. In another research, *X. campestris* PTCC 1473 showed a maximum XG yield of 8.9 g/L from a reducing sugar concentration of 21.5 g/L using acid-hydrolyzed broomcorn stem [139].

### 5.4. Substrate Optimization Strategies

In order to promote green production of XG, the utilization of waste from various food and industrial waste as a potential substrate for *X. campestris* fermentation requires some optimization. This necessity arises from the heterogeneous composition and imbalanced nutrients of this waste-derived substrate, which often leads to variability in the yield and quality of XG. Hence, effective optimization is essential in terms of compositional adjustment, pretreatment, and hydrolysis, and the integration of advanced statistical design of experiment (DoE) and artificial intelligence (AI) modelling to produce a more predictive and effective XG production system.

As studied by Dos Santos et al. [138], not all agricultural residues are capable of generating a sufficient amount of carbon source for XG production due to a lack of micronutrients. However, its capabilities as substrates vary significantly depending on their origin, operating process, and storage conditions that might affect their carbon, nitrogen, and micronutrient composition. This instability may alter microbial metabolism and affect the XG production and its rheological properties.

Therefore, strategic identification followed by targeted supplementation for compositional balancing of the C/N ratio and micronutrients is vital to increase the metabolism for XG production. This step is much better than the full replacement of synthetic media in a cost-effective manner. Agricultural, industrial, or food processing residues, for example, the jackfruit seed powder that is high in carbon but insufficient in nitrogen and micronutrients like peptone, phosphate salt, and citric acid. After the addition of these limiting elements, a significant XG production was recorded [90]. By providing minimal quantities of the limited elements, the overall performance of the substrate can be enhanced without increasing production cost while pursuing an environmentally friendly step. This step can be further enhanced and optimized systematically using statistical methods such as RSM to fine-tune the nutrient interactions, which provide predictive insight into the most effective nutrient balance approaches. This might strengthen the process predictability and promote the utilization of waste substrates.

Due to the complex and recalcitrant properties of lignocellulosic wastes, *X. campestris* often faces difficulties in metabolizing the nutrients required for the XG biosynthesis [140]. Consequently, systematic pretreatment for these substrates is crucial to break down the rigid molecular matrix into a simpler structure that is more accessible for microbial utilization. Pretreatment can be classified into physical, chemical, physicochemical, and biological methods. Physical pretreatments, such as milling or grinding [141], reduce particle size and increase surface area, thereby improving enzymatic hydrolysis and microbial accessibility to fermentable sugars. Physicochemical methods, including steam explosion and liquid hot water treatment [142], combine thermal and pressure effects to solubilize hemicellulose and partially depolymerize lignin, resulting in higher sugar release without excessive chemical usage. However, chemical pretreatment using acids such as sulfuric acid (H_2_SO_4_) is widely employed to hydrolyze the hemicellulose and partially solubilize lignin to enhance the release of fermentable sugars [143]. The drawback of this step is contributed by excessive use of chemicals that might generate inhibitory compounds that will impact the quality and quantity of XG production [144]. Hence, optimization of pretreatment parameters such as the concentration and the reaction time should be controlled to ensure maximum sugar recovery with minimal or negligible inhibitor formation.

Alternatively, biological pretreatment using bio-enzymes or microorganisms could be an eco-friendly approach since it could work under mild environmental conditions to degrade the lignin and hemicellulose while preserving the release of sugars for fermentation [145]. Although this process is much slower than the chemical approaches, it could reduce the consumption of chemicals and overall production cost. This can be further optimized by integrating mild chemical treatment with an enzymatic or microbial approach to maximize substrate conversion and achieve higher XG yield.

Other than that, the use of statistical and artificial intelligence (AI) tools such as RSM can also be employed to optimize the interactive effects among process variables such as pH, temperature, agitation rate, and inoculum size for the maximum XG production. For example, RSM was used by Ramos et al. [18] for optimizing XG production using alternative sugar of demerara. The results showed an optimized condition of 30 g/L demerara sugar, 1 g/L of MgSO_4,_ and 1 g/L of K_2_HPO_4_, yielding 1.3839 g/L of XG. RSM was also used by Mohsin et al. [127], resulting in 30.19 g/L of XG after 1.62% acid hydrolysis with 85% carbon source from orange peel hydrolysate and 30.4 °C temperature. Similarly, there are some other studies that applied Artificial Neural Network (ANN) modelling to fine-tune the fermentation parameters and predict the XG production. ANN accurately predicts the apparent viscosity of XG by using concentration, derivatives (0.5% or 1%), molecular weight, degree of substitution, and shear rate [146]. ANN also accurately predicts XG production (with only 0.12% error) by putting sugar, peptone, KH_2_PO_4_, (NH_4_)_2_SO_4_, and FeCl_3_ concentrations [106].

## 6. Challenges and Future Prospects

### 6.1. Technical Barriers and Research Gaps

Despite significant advancements in XG production, several technical challenges continue to limit the scalability and sustainability of newer approaches. One of the main barriers is the efficient utilization of alternative substrates, especially those derived from lignocellulosic biomass or agro-industrial waste. These substrates often require extensive pretreatment to make sugars bioavailable for fermentation, which can introduce inhibitory by-products and increase overall processing costs [147]. Another key challenge is the low yield and productivity often observed when using non-conventional substrates. This may be attributed to the limited metabolic capacity of *X. campestris* to metabolize complex carbon sources [148]. Additionally, strain instability, variability in substrate composition, and incomplete understanding of metabolic fluxes contribute to inconsistent production outcomes.

The downstream processing of XG, particularly extraction and purification, remains resource-intensive [4]. Many green extraction technologies are still in their infancy and lack the robustness and scalability required for industrial deployment. Moreover, limited integration of omics data and metabolic engineering tools in strain improvement strategies has slowed progress in optimizing XG biosynthesis.

### 6.2. Regulatory and Safety Considerations

As XG is widely used in the food, pharmaceutical, and cosmetic industries, regulatory compliance is crucial. Any transition toward new substrates, particularly those derived from waste, must ensure biosafety, quality control, and contaminant removal. Regulatory agencies may impose restrictions or require extensive validation before approving such products for human consumption [149]. The use of genetically modified organisms (GMOs) in XG production also presents regulatory hurdles [150]. While genetic engineering offers tools to enhance yield and functionality, the adoption of genetically improved strains may face resistance in certain markets due to labeling requirements and public perception. In addition, standardization of substrate sources, particularly agricultural residues, remains a challenge for ensuring consistent product quality across production batches.

### 6.3. Innovations in Bioprocessing and Genetic Engineering

Recent innovations in bioprocess intensification and genetic engineering are opening new pathways for sustainable XG production. These include the development of engineered strains of *X. campestris* or alternative microbial hosts with improved substrate utilization, stress tolerance, and higher biosynthetic capacity [151]. Advancements in systems biology, CRISPR-Cas technologies, and metabolic flux analysis allow for precise modifications to the biosynthetic pathway, enhancing yield and reducing by-product formation [152]. Recent metabolic engineering efforts in *X. campestris* have focused on modifying genes within and outside the gum operon to enhance xanthan yield and tailor polymer properties. Overexpression of gum cluster (*gumB*–*gumM*) has been shown to increase the initiation rate of repeat-unit assembly [153], thereby improving overall polysaccharide productivity, while also enabling adjustment of rheological properties such as viscosity, gel strength, and thermal stability [55]. Modulation of Wzy *polymerase* influences chain length and molecular weight distribution [154].

Beyond the gum cluster, engineering of precursor supply pathways, such as genes involved in UDP-glucose, GDP-mannose, and UDP-glucuronic acid biosynthesis (e.g., *pgm*, *galU*, *ugd*, and *manC*), has been proposed to increase carbon flux toward nucleotide sugars and reduce by-product formation [155,156,157]. CRISPR-Cas-assisted editing also offers potential for knocking out competing pathways that divert carbon to cell biomass rather than exopolysaccharide production [158]. In addition, introducing or upregulating genes related to pentose utilization and lignocellulosic sugar metabolism could improve the direct use of hydrolysates from agricultural residues [159]. Meanwhile, the integration of continuous fermentation systems, membrane-based separation, and low-energy extraction technologies can significantly lower the environmental footprint of the process.

### 6.4. Outlook for Sustainable XG Industry

Looking forward, the sustainable production of XG is expected to evolve through the convergence of biotechnology, process engineering, and environmental science. The continued exploration of renewable and waste-based substrates aligns well with circular economy principles and could significantly reduce dependency on food-grade sugars. To realize this potential, multidisciplinary efforts are needed to optimize microbial strains [63], develop efficient substrate pretreatment protocols [16,160], and implement eco-friendly extraction methods [15,161]. Moreover, collaboration between academia, industry, and regulatory bodies will be essential to ensure that innovations are translated into commercially viable and safe products.

## 7. Conclusions

Sustainable XG production requires coordinated advances across substrate valorization, fermentation efficiency, and environmentally responsible downstream processing. The increasing use of agro-residues, industrial by-products, and lignocellulosic biomass demonstrates clear potential to reduce dependence on refined sugars while simultaneously addressing organic waste management challenges. When combined with optimized microbial strains, controlled aeration strategies, and low-energy recovery techniques such as membrane separation and electro-dewatering, these approaches can substantially lower solvent consumption, process emissions, and overall production costs.

Looking forward, the integration of bioprocess intensification, metabolic systems engineering, and real-time process control will be critical for translating laboratory-scale innovations into industrially viable platforms. In particular, genetic engineering tools that enhance substrate flexibility and inhibitor tolerance can enable the direct conversion of heterogeneous waste streams into high-value biopolymers. Coupling these biological advances with continuous processing, solvent recycling, and water reuse will allow the development of closed-loop production systems. From a circular bioeconomy perspective, XG production can evolve from a linear fermentation model into a resource-recovery platform in which food processing residues, agricultural waste, and industrial effluents are converted into functional hydrocolloids with minimal material loss.

Despite these advances, several limitations remain, including the variability of waste-derived substrates, the lack of standardized pretreatment and compositional characterization, and the limited pilot-scale validation of emerging solvent-free recovery technologies. In addition, the techno-economic feasibility and long-term operational stability of membrane and electro-assisted processes under high-viscosity conditions are still insufficiently demonstrated. Addressing these gaps through integrated scale-up studies and life-cycle assessment will be essential for translating laboratory findings into industrially viable and circular XG production systems.

Future research should therefore prioritize (i) standardized characterization of waste-derived substrates; (ii) techno-economic and life-cycle assessments of emerging extraction technologies; (iii) scalable hybrid processes that combine biological and membrane-based separations. These efforts will be essential to ensure product safety, regulatory compliance, and consistent quality for food and pharmaceutical applications. Ultimately, advancing XG production within a circular bioeconomy framework offers a pathway toward low-carbon, waste-valorizing, and economically resilient biopolymer manufacturing.

## Figures and Tables

**Table 1:** Influence of strain type, carbon and nitrogen sources, and operating conditions on XG production.

Strain	Inoculum Size (*v*/*v*)	Media Composition	Fermentation Condition	XG Yield (g/L)	References
*X. axonopodis* pv. *mangiferaeindica*	10%	C: Glycerol, maltose N: Peptone, yeast extract	pH: 7 Temperature: 26 °C Speed: 180 rpm Time: 120 h	1.46	[70]
*X. campestris* 260	One loop	C: Glucose, maltose N: Peptone, yeast extract	pH: 6.4–6.8 Temperature: 30 °C Speed: 200 rpm Time: 24 h	15.42	[76]
*X. campestris* 2956	10%	C: Glucose, maltose extract N: Peptone, yeast extract	pH: 6.4–6.8 Temperature: 30 °C Speed: Constant Time: 24 h	4.8	[77]
*X. campestris* B6720	5%	C: Glucose N: Yeast extract Extra: KH_2_PO_4_, K_2_HPO_4_, MgSO_4_·7H_2_O	pH: 6.4–6.8 Temperature: 28 °C Speed: 250 rpm Time: 72 h	8.84	[78]
*X. campestris* CCTCC M2015714	10%	C: Glycerol, glycerides, methanol N: Yeast extract, peptone Extra: NaNO_3_, FeSO_4_, MgSO_4_, KH_2_PO_4_, K_2_HPO_4_	pH: 7 Temperature: 30 °C Speed: 600 rpm Time: 60 h	11	[79]
*X. campestris* PTCC1473	5%	C: Glucose, citric acid N: NH_4_NO_3_ Extra: KH_2_PO_4_	pH: 7.2 Temperature: 30 °C Speed: 200 rpm Time: 48 h	7.8	[80]
*X. campestris* LRELP-1	10%	C: Glucose, fructose, sucrose, lipids N: Crude protein, amino acids	pH: 7 Temperature: 30 °C Speed: 300 rpm Time: 120 h	11.73	[24]
*X. campestris mangiferaeindicae* 2103	20%	C: Glycerol, glycerides, lipids N: Urea Extra: KH_2_PO_4_, antifoam	pH: 7 Temperature: 28 °C Speed: 498 rpm Time: 120 h	5.59	[81]

**Table 2:** Comparison of Advanced XG Extraction Technologies.

Technology	Solvent Use *	Energy Demand *	Product Purity *	Polymer Integrity	Scalability	Development Stage	Key Advantages	Main Limitations
Ultrasound-assisted Extraction (UAE)	Low	Moderate	Moderate–High	Risk of chain degradation at high power	Limited continuous scale	Lab to Pilot	Faster extraction, lower temperature, reduced solvent	Energy distribution, viscosity handling
Pulse Electric Fields (PEF)	Very low	Low–Moderate	High	Preserved molecular weight	Pilot	Lab to Pilot	Non-thermal, rapid cell disruption	High CAPEX, electrode fouling, conductivity limits
High Voltage Electrical Discharge (HVED)	Low	High	Moderate	Possible oxidative depolymerization	Low	Lab	No chemical additives, effective disruption	High energy, radical formation, scale-up unknown
Membrane filtration	None	Low–Moderate	High	Preserved	High (with fouling control)	Pilot to Semi-industrial	Continuous operation, high purity, solvent-free	Fouling, viscosity, cleaning cost
Electro-dewatering/electrofiltration	None	Low	High	Preserved	Moderate	Pilot to Semi-industrial	Lower drying energy, improved dewatering	Electrode corrosion, conductivity sensitivity
Foam fractionation	None	Very low	Low–Moderate	Preserved	Low	Lab	Minimal energy and solvent	Low efficiency for XG, surfactant requirement

* Expressed as qualitative comparative indicators based on reported trends in previous studies [119,120,121,122,123,124].

**Table 3:** Production of XG with alternative carbon and nutrient sources.

Strain	Inoculum Size (*v*/*v*)	Media Composition	Fermentation Condition	XG Yield (g/L)	References
*X. campestris*	10%	C: Orange peel hydrolysate, citric acid N: Yeast extract Extra: K_2_HPO_4_, MgSO_4_, H_3_BO_3_, ZnCl_2_, FeCl_3_, CaCO_3_	pH: 7 Temperature: 30 °C Speed: 300–400 rpm Time: 96 h	30.19	[127]
*X. campestris* ATCC 13951	10%	C: Winery wastewater Extra: CaCO_3_	pH: 6.5–7.5 Temperature: 25 °C Speed: 200 rpm Time: 96 h	24.18	[113]
*X. campestris* MTCC 2286	5%	C: Acidified and aerated sugarcane molasses (AASM) N: Yeast extract Extra: K_2_HPO_4_, MgSO_4_	pH: 7 Temperature: 30 °C Time: 48 h	12.23	[128]
*X. campestris* NCIM 2961	5%	C: Jackfruit seed powder (JSP), peptone N: Peptone Extra: Citric acid, KH_2_PO_4_, K_2_HPO_4_	pH: 7 Temperature: 37 °C Speed: 120 rpm Time: 72 h	51.62	[90]
*X. campestris* NRRL B-1459	5%	C: Glucose (date-juice) N: (NH_4_)_2_SO_4_	pH: 7.0 Temperature: 28 °C Speed: 180 rpm Time: 48 h	24.5	[129]
*X. campestris* PTCC1473	5%	C: Date extract N: NH_4_NO_3_ Extra: H_3_BO_3_, MgCl_2_, Na_2_SO_4_, ZnO, FeCl_3_·6H_2_O, CaCO_3_, FeSO_4_, HCl	pH: 7.0 Temperature: 28 °C Speed: 200 rpm Time: 72 h	11.2	[91]
*X. campestris* PTCC1473	5%	C: Date extract N: (NH_4_)_2_SO_4_ Extra: KH_2_PO_4_	pH: 7.0 Temperature: 30 °C Speed: 395 rpm Time: 48 h	6.51	[1]
*X. pelargonii* PTCC1474	5%	C: Lactose (cheese whey) N: (NH_4_)NO_3_ Extra: KH_2_PO_4_, MgSO_4_ 7H_2_O, citric acid, H_3_BO_3_, ZnCl_2_, FeCl_3_, CaCl_2_	pH: 7.0 Temperature: 28 °C Speed: 250 rpm Time: 48 h	12.8	[130]

## Data Availability

The original contributions presented in this study are included in the article. Further inquiries can be directed to the corresponding author.
